# FOXA2 controls *Pkd1l1 *expression in the mouse node during left-right determination

**DOI:** 10.1186/2046-2530-4-S1-P37

**Published:** 2015-07-13

**Authors:** D Leordean, D Grimes, J Keynton, J Maier, B Harfe, M Benson, A Gray, S Bhattacharya, D Norris

**Affiliations:** 1 MGU, Cilia, Development and Disease, MRC Harwell, Didcot, UK; 2 Department of Molecular Genetics and Microbiology, College of Medicine, University of Florida, Gainesville, FL, USA; 3 Radcliffe Department of Medicine, University of Oxford, Oxford, UK

## Objectives

The embryonic node is a ciliated pit-like structure that is important in left-right (L-R) determination. *Pkd1l1*, encoding a membrane-bound ciliary protein, is expressed specifically in the node and is required to establish normal L-R patterning. Given the importance of this gene, we set out to understand its regulation.

## Methods

*Pkd1l1 *and *FoxA2 *expression was compared through whole-mount *in situ *hybridization and β-galactosidase staining in 7.5-9.5 dpc mouse embryos. Bioinformatic analysis was used to characterise FOXA2 binding regions upstream of *Pkd1l1 *in the human-mouse lineage. Finally, luciferase assays were used to test the functionality of these potential regulatory regions.

## Results

Our expression studies indicate that a regulatory relationship might exist between FOXA2 and *Pkd1l1*. Furthermore, *Shh^-/- ^*and *FoxA2^c/c^*;*ShhcreER^T2 ^*embryos display abnormal *Pkd1l1 *expression. Indeed, previous studies identified FOXA2 binding regions upstream of *Pkd1l1*, in both human and mouse. Our work has revealed the two regions to be non-homologous, but conserved in the human-mouse lineage, in some cases to the binding site level. Although bioinformatic analysis predicts lower binding affinities for the conserved mouse-identified region in human and *vice versa*, luciferase assays indicate that the conserved regions are capable of driving gene expression.

## Conclusion

Our study implicates FOXA2 in the regulation of *Pkd1l1 *and characterises two FOXA2 binding regions upstream of this gene. Both regions are conserved across the human-mouse lineage to varying degrees. Furthermore, in both mouse and human, the two regions are able to activate gene expression.

